# Evaluating the Evidence for Lymphatic Filariasis Elimination

**DOI:** 10.1016/j.pt.2019.08.003

**Published:** 2019-11-01

**Authors:** Emma L. Davis, Lisa J. Reimer, Lorenzo Pellis, T. Deirdre Hollingsworth

**Affiliations:** 1 University of Warwick, Coventry CV4 7AL, UK; 2 Liverpool School of Tropical Medicine, Pembroke Place, Liverpool L3 5QA, UK; 3 University of Manchester, Oxford Road, Manchester M13 9PL, UK; 4 Big Data Institute (BDI), Oxford University, Old Road Campus, Oxford OX3 7LF, UK

## Abstract

In the global drive for elimination of lymphatic filariasis (LF), 15 countries have achieved validation of elimination as a public health problem (EPHP). Recent empirical evidence has demonstrated that EPHP does not always lead to elimination of transmission (EOT). Here we show how the probability of elimination explicitly depends on key biological parameters, many of which have been poorly characterized, leading to a poor evidence base for the elimination threshold. As more countries progress towards EPHP it is essential that this process is well-informed, as prematurely halting treatment and surveillance programs could pose a serious threat to global progress. We highlight that refinement of the weak empirical evidence base is vital to understand drivers of elimination and inform long-term policy.

Highlights

The current target of elimination as a public health problem (EPHP) for lymphatic filariasis was originally devised with the intention of interrupting transmission. However, some countries that have achieved EPHP are still finding new cases.

Analysis of the evidence for key biological determinants suggests that a target threshold of <1% microfilaria (mf) prevalence is not likely to be sufficient for transmission interruption in communities with a mid-to-high annual biting rate.

The experimental evidence underlying estimates is insufficient or inconsistent, particularly transmission rates from vector to human, leading to high uncertainty in confidence of elimination success.

Local biting rate is expected to be highly variable between settings and could have a large impact on elimination feasibility for a given target prevalence.

Further experimental studies are needed to refine our understanding of LF elimination thresholds.

## Global Situation and Progress

There are currently 886 million people across 52 countries worldwide at risk of LF^i^. Infection is caused by a mosquito-transmitted filarial worm and, if left untreated, can lead to permanent and debilitating disability. The Global Program to Eliminate Lymphatic Filariasis (GPELF) set a target of **elimination as a public health problem (EPHP)** (see Glossary) in 1997, leading to over 7.1 billion treatments delivered as part of **mass drug administrations (MDAs)** since 2000^i^. In 2011, the WHO published guidelines for halting treatment and verifying EPHP through the use of **transmission assessment surveys (TAS)** to measure a target threshold^ii^,iii .By October 2018, 14 countries had reached this target, and 554 million people worldwide no longer require mass treatments^iv^.

As indicated by the name of the TAS, it was hoped that reaching these targets would lead to elimination of transmission (EOT) in most areas. However, in Sri Lanka the TAS has been demonstrated as not sensitive enough to detect low-level persistence [1,2], and pockets of transmission are still being found despite EPHP validation. The community is now revisiting the TAS methods, including the original target of 1% **microfilaria (mf)** prevalence [3], particularly in the context of the new **triple-drug** regimen which is hoped to accelerate progress, but will require different post-treat-ment surveillance [4].

It is possible that achieving EPHP, according to the current definition, will lead to EOT in some settings [5,6], but the high levels of variability between localities, and uncertainty in our knowledge of transmission, make it hard to predict where this will occur. This is exacerbated further by seasonal variation in environmental conditions, which has been shown to impact a number of helminth infections [7,8]. Residual infection remaining after MDA cessation can lead to resurgence and reintroduction [9,10], with long-term persistence dependent on a range of factors [11].

## Sexual Reproduction in the Host, and Elimination

The sexual reproduction of filarial worms requires both male and female parasites to be present in an individual host for microfilariae production, so at a sufficiently low prevalence we would expect mostinfections to be nontransmissible due to low parasite load (i.e., a low probability of male and female adults in the same host). This is expected to result in fewer onward infections, and hence increasingly lower prevalence and intensity, until infection dies out. The threshold below which we expect this phenomenon to occur is called the **breakpoint [12,13]**. As the focus of some neglected tropical disease (NTD) programs has shifted from control towards elimination, there have been a number of studies aiming to quantify these thresholds for a variety of helminth infections within the NTD umbrella [14–17].

**Figure 1:**
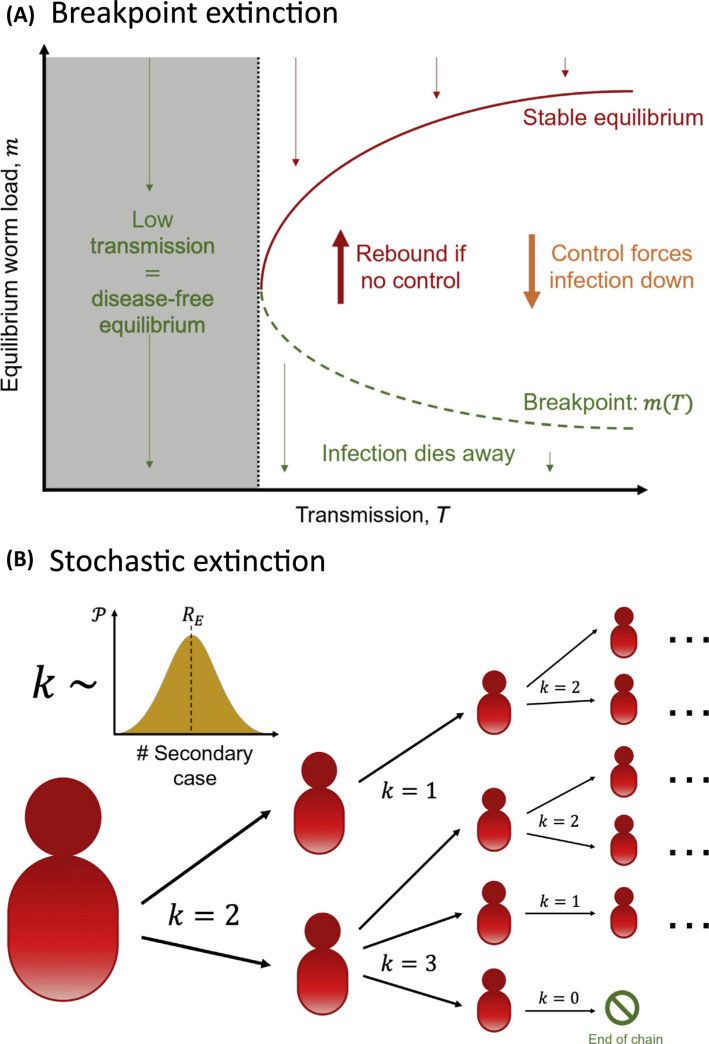
Lymphatic Filariasis Extinction Theory. Schematics comparing the theory behind breakpoint extinction (A) and stochastic extinction (B) for lymphatic filariasis. (A) For sufficiently low transmission intensities (i.e., low biting rates), disease levels will drop away to zero. Beyond the critical transmission level (black broken line) there are three equilibria: high disease (stable, red), low disease (unstable ‘breakpoint’, green), disease-free (stable, black). Disease levels above the breakpoint will increase to the higher equilibrium, whereas disease levels below will decrease to zero. (B) Visualdepiction of a branching process starting with one infectious individual. The number of secondary infections caused by each currently infectious individualare sampled from th secondary case distribution. This is used to simulate the onward chains of infection; extinction occurs when all chains die out (i.e., have no secondary cases). Stochastic variation can cause this to occur even above the theoretical breakpoint threshold.

This theory has certain consequences for control (Figure 1A). If transmission is sufficiently low, then the infection is expected to die out. If there is a higher transmission rate, outcomes depend on the mean worm load in the population; if, usually through control strategies, the worm load is below the green broken line (the breakpoint) then elimination is assured. Previous modelling studies that have assessed breakpoint thresholds have found values of much less than 1% mf prevalence [10,18–20]. It has been previously demonstrated that factors such as parasite aggregation and vector competence will further affect these thresholds [21], and the majority of studies have focused on specific geographical areas, resulting in a wide range of suggested breakpoints across the literature.

Glossary

**Annual biting rate (ABR):** the average number of mosquito bites per person per year.

**Basic reproductive number (*R*_0_):** the average number of new infectious cases generated by one infectious case in an entirely susceptible population.

**Blood feeding rate (BFR):** the rate at which mosquitoes take a blood meal.

**Branching process:** a stochastic process which consists of collections of random variables, which are indexed by the natural numbers (1,2,3,...).

**Breakpoint:** a prevalence level below which sustained transmission is not viable and elimination (zero cases) becomes an absorbing state.

**Effective reproductive number (*R*_e_):** the average number of new infectious cases generated by one infectious case in a population made up of both susceptible and infectious hosts.

**Elimination as a public health problem (EPHP):** as measured by TAS, a metric used by the WHO to validate programme success. Intended to naturally lead to EOT.

**Extrinsic incubation period (EIP):** the time it takes for ingested mf to develop to infectious L3 larvae in the mosquito.

**Implementation unit:** the designated level of the administrative unit in a country, for which the decision to administer antifilarial drugs to the entire population is taken if it is identified as having indigenous transmission or endemicity.

**L3:** the third larval stage of the parasite; at this point it is infectious to humans.

**Mass drug administration (MDA):** the administration of drugs to a whole population, irrespective of disease status.

**Microfilaria (mf):** developmental stages in the bloodstream, produced by fertilized female worms, that can be picked up by mosquitoes.

**Transmission assessment surveys (TAS):** a series of surveys designed by the WHO to measure post-MDA infection levels and verify EPHP.

**Triple drug:** ivermectin and diethylcarbamazine and albendazole (IDA): a drug combination that has recently become the gold standard for treatment of LF.

**Univariate:** literally ‘of one variable’. Univariate analysis explores variables one-by-one, keeping all others fixed.

**Vector-host ratio:** the number of vectors per human in a geographicalregion.

Measuring breakpoints that are substantially lower than 1% mf prevalence would require infeasible sample sizes and survey costs. In this review we do not argue for a specific breakpoint, instead focusing on asserting that the experimental evidence is too uncertain to conclusively support a 1% threshold and emphasizing the importance of spatial heterogeneity.

Whilst breakpoint theory is extremely useful, it is also possible for stochastic, or chance, extinction to occur before this breakpoint is reached, particularly when infection levels are low (Figure 1B). The probability of elimination, given a particular prevalence (e.g., 1%), can be calculated by considering the probability that a chain of transmission will die out (in mathematics we call this chain a **branching process [22]**). These types of branching process methods have been used for soil-transmitted helminths [23,24], but have been adapted here to account for vector-borne transmission with an aggregated bite risk [25,26].

Current guidelines mean that EPHP is validated after passing TAS, but we have little experience in what this means for long-term transmission. Assuming for simplicity that TAS is able to measure a true mf prevalence of less than 1%, this theory of stochastic extinction can be used to estimate how the future probability of EOT (zero cases) depends on a range of setting- and disease-specific variables. This process uses the distribution of the number of infectious secondary cases caused by one infectious individual, the mean of which is the **effective reproductive number** (***R*_e_**).

**Figure 2:**
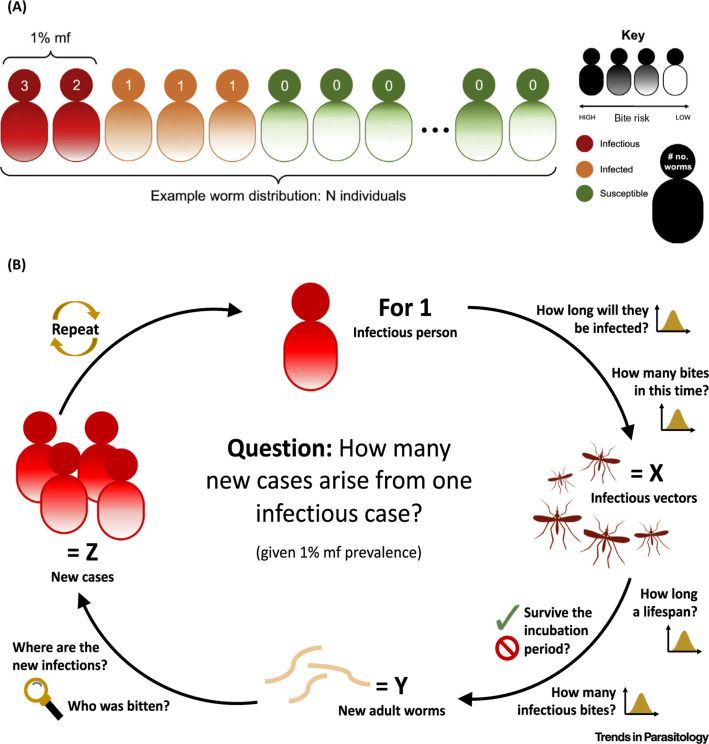
Simulating Branching Process Extinction. A schematic describing the simulation process for calculating the number of secondary cases produced by one infectious individual in a population with 1% microfilaria (mf) prevalence. (A) Allocate distribution of adult worms and bite risks across the population. Individuals with 1 worm are infected but are not infectious, individuals with two or more worms are considered potentially infectious. (B) Generational calculation of number of new infectious cases caused by one infectious individual. One infectious individual infects X vectors. The vectors that survive the incubation take infectious blood meals, resulting in Y new adult worms. These worms are distributed across the population according to bite risk aggregation, resulting in Z new infectious (≥2 worms) individuals.

As a toy example, for a population of 1000 and 1% mf prevalence, we consider a distribution of individual worm burdens (Figure 2A). Infections with only one worm are nontransmissible. From one infectious person you then get the number of new cases, Z, caused during their infectious period (Figure 2B). Since transmission represents a chance event, Z is best represented by a distribution, and acts as a proxy for *R_e_.* This distribution determines the probability of the transmission chain dying out, that is, no further cases, at some point in the future; for more detail see Box S1 in the supplemental information online. We use this to give a **univariate** demonstration of the present parameter uncertainty and how this might impact two epidemiological measures: the probability of elimination and the effective reproductive number.

## Empirical Evidence for Life-Cycle Variables

**Figure 3:**
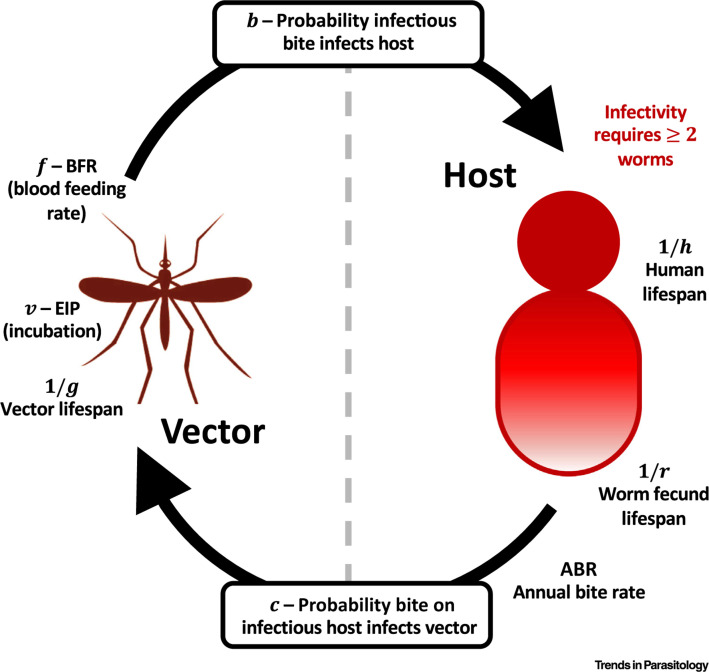
Lymphatic Filariasis Life-cycle. Life-cycle schematic demonstrating key biologicalvariables that could affect prediction of elimination success. Duration of infection is determined by human and fecund worm lifespans. Infection from host to vector depends on the annualbiting rate (ABR) and the probability that a bite on an infectious host infects a vector. The number of vectors that survive to infectivity depends on the extrinsic incubation period (EIP) and vector lifespan. Transmission from vector to host is then determined by the blood feeding rate and the probability that an infectious bite results in a viable adult infection, as wellas the requirement for two or more worms for infectivity.

We now review evidence for key parameters in the life cycle which drive transmission (Figure 3). As previously mentioned, a number of these variables, such as the **annual biting rate (ABR)**, are likely to introduce large differences due to the high spatial variability. Others, such as the probability an infectious mosquito bite results in a viable human infection, have the potential to be more consistent across settings, but currently lack in experimental evidence.

A detailed literature review turns up widely varying estimates of ABR, partially due to geographical variation. These values, from countries with a history of LF endemicity, range from three [27] to 611

[28] bites per person per day. A number of these are based on human landing catches [27–29], with the majority relying on studies from the 1960s and 1970s [28], whilst some are derived from models [30]. Despite a wealth of historic studies, supported by the malaria literature, human landing catches are often considered unethical and give highly variable results. Relying on historic estimates can also disregard changes in socioeconomic conditions resulting in decreased vector-human contact.

Current estimates in the literature of the **basic reproductive number**, *R*_0_, range from zero to 2.5 [31], depending on the **vector-host ratio** (an alternative metricto ABR). Although setting-specific values of *R*_0_ for other diseases can often be calculated from infection data, the global landscape of public health history for LF means that we have very little contemporary baseline (precontrol) data with which to do this. As an alternative, we can consider the previously mentioned estimation of *R*_e_.

Another important, but largely uncertain, factor is the degree of parasite aggregation, measured inversely by the negative binomial *k*. For LF, adult worm aggregation is considered to be driven by heterogeneous transmission, caused by host variation in bite risk [15]. Initial estimates for k were based on mf data (*k* = 0.08, 0.3 [21,26]). However, a recent study in Papua New Guinea used bite and mf data to demonstrate that the *k* for bite risk is an order of magnitude larger than that for mf aggregation, giving a refined estimate of 0.73 (standard deviation 0.035), with site-specific estimates ranging from 0.3 to 1.3 [15,26]. We will now separate transmission into two parts: humans to mosquitoes, and mosquitoes to humans. When considering the former, the key variables are duration of infection, which depends on fecund worm lifespan, and the probability that a vector biting an infected host will become infectious.

Often worm lifespan is stated as being 6–8^i^ or 5–10 years [32,33], but reference trails rarely reveal empirical evidence. There are studies that corroborate similar ranges, such as 2.1–5.4 [34] or 9.1–11.8 [35] years, but there are also estimates in the literature of up to 40 years [36].

Infectivity to mosquitoes depends on mf intensity, leading to wide ranges of 15–60% of vectors becoming infected from a single mf-positive bite [37,38].

Infection from vector to human is governed by the number of infectious bloodmeals one mosquito will take – calculated from vector survival and competence, **extrinsic incubation period (EIP)** and **blood feeding rate (BFR)** – and the probability one infectious bite will result in a viable infection. There are reasonable estimates for vector survival and BFR from the malaria literature [27,39] and for LF incubation [40], although these do not typically account for the impact of infection on survival [37].

One key parameter of infection, the probability an infectious bite results in a mature human infection, is largely unknown. Estimates range from 10^–5^ to 10^–3^ [41,42] and are usually broken down into three steps: the **L3** leaving the vector, entering the host, and developing to fecundity. The first step is relatively straightforward to measure [43], although it poses ethical issues, and the second can be estimated using mouse models [44,45]. The third is harder; best estimates are calculated by using *Brugia malayi*studies to derive a daily death rate and then applying this across the longer *Wucheria bancrofti* developmental period [32,46].

## Quantifying the Probability of Elimination

**Figure 4:**
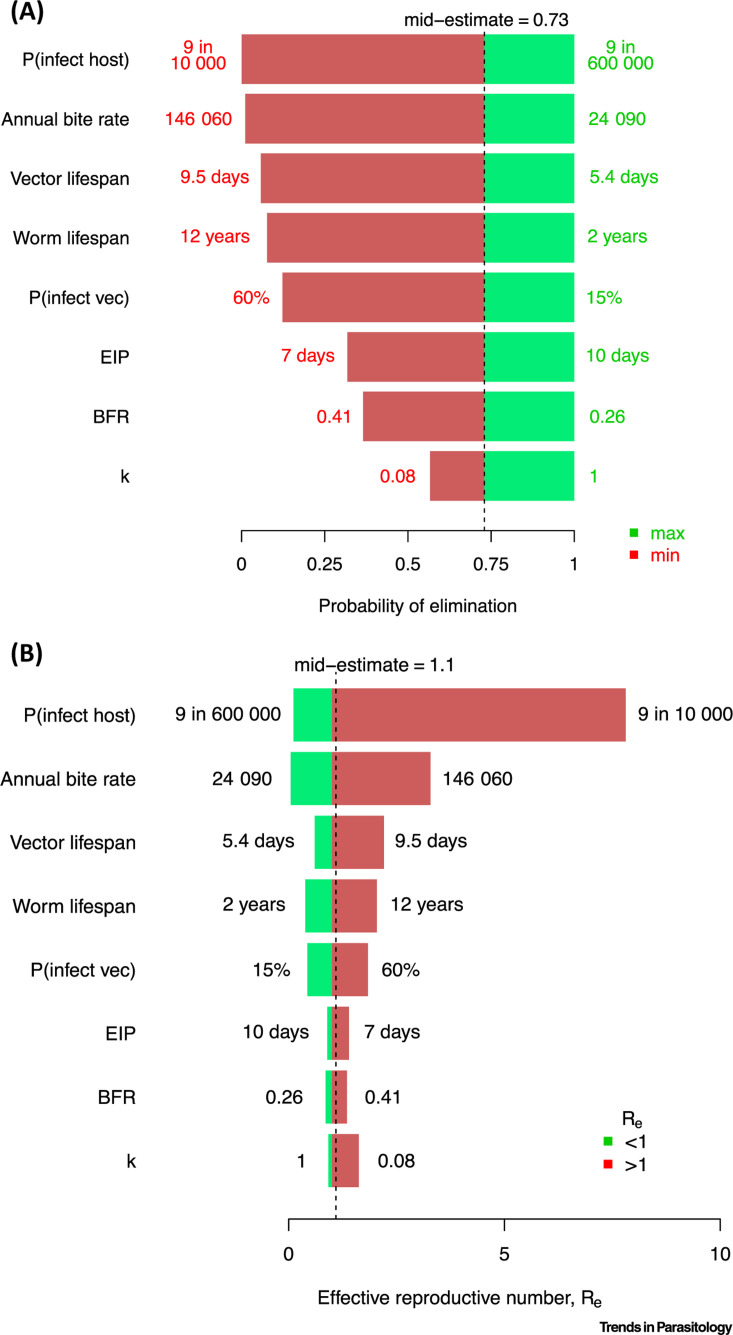
Predicting Elimination Probabilities. Illustration of the potential impact of high uncertainty in variables by considering their univariate impact on the probability of elimination (A) and the effective reproductive number (B) for the key biological variables of the lymphatic filariasis (LF) transmission cycle, assuming a microfilaria (mf) prevalence of 1% and a human population size of 1000. References for ranges of variables considered can be found in Table S1 in the supplemental information online. Note that this univariate analysis should be interpreted carefully as variables are likely to be correlated in ways which we cannot yet account for. For example, the mid-estimates here have been chosen to represent a mid-ground of ranges found in the literature and are not necessarily representative of the true values or ranges that may exist across real-world settings. Abbreviations: BFR, blood feeding rate; EIP, extrinsic incubation period.

If we include these parameters in the simple framework described above, we can see how the uncertainty affects our estimates of key epidemiological measures (Figure 4). The mid-points of elimination probability (0.73) and *R_e_* (1.1) are not intended to be true estimates, rather they represent a midground of the parameter ranges found in the literature and a basis for comparison.

The variable which generates the most univariate uncertainty is the probability that an infectious mosquito bite will infect a human, b, due to the wide range of possible values. Variation in elimination probability due to ABR, which is correlated with the basic reproductive number (R_0_), is also very high. This is due to both measurement inaccuracy and spatiotemporal variability. Parameters that are known to be key drivers in the probability of elimination, worm fecund lifespan and the degree of adult worm aggregation [21,47,48], potentially induce lower uncertainty here due to considering narrower plausible intervals.

In addition to the probability of elimination, we also consider the effective reproductive number, *R_e_.* It is important to note that, for helminth infections, metrics often refer to the number of adult filarial worms arising from one adult filarial worm, rather than considering human cases. However, the theory is similar enough to allow heuristic comparison. Our mid-estimate for *R_e_* is chosen to be close to 1, representative of the low-level transmission observed in some post-MDA settings, but varying the probability that an infectious mosquito bite will lead to a patent infection (*b*) can lead to an order of magnitude difference. In fact, it is possible to push the estimate of *R_e_* across the critical threshold (*R_e_* = 1) between extinction and endemicity by adjusting any variable within the ranges found in the literature. This reinforces the importance of using reliable variable estimates when making predictions, particularly in elimination settings where infection data are sparse.

## Recommendations

Due to the demonstrated uncertainty that knowledge gaps, particularly in the establishment of a patent infection, can cause in estimating elimination thresholds it would be prudent to refine the evidence for these variables. Here we discuss a few options for future studies and analyses that we believe could strengthen the knowledge base.

The probability that an infectious bite leads to an infectious host cannot be measured experimentally in humans; however, we can improve current estimates with anecdotal and observational studies. Longitudinal studies can provide evidence of the time to antigen positivity and the time to microfilaria positivity in children, or in adults that have moved from nonendemic to endemic regions. One existing study, looking at acquisition in travelers, surmises that the majority of cases are in individuals who spent in excess of 6 months in an endemic region [49], whereas another cites a number of travelers contracting infection with only 1 month of exposure [42]. Entomological studies routinely estimate ABR through human landing catch data, and individual exposure can be quantified based on net usage and vector biting habits [50,51].

Outstanding Questions

How can we translate our understanding of elimination dynamics to clear and feasible guidelines for public health programs?

Is there a universal threshold, or do we need to tailor predictions for different communities and settings?

What are the key determinants that vary between settings, and how can we measure them?

How can we reliably measure annual biting rate for different settings?

How can we refine our estimates of transmission probability from vectors to humans?

How can we determine where 1% mf prevalence is a threshold below which elimination is likely?

If lower target thresholds are required for elimination of transmission, then are we realistically able to measure these using current tools?

Whilst we can measure that prevalence is below certain thresholds, is this sufficient evidence of elimination of transmission?

In settings where we are still seeing new cases after EPHP verification, what is the probability of large-scale resurgence?

What is a suitable survey design in a context of limited resources?

How will the new diagnostic affect elimination measurement?

How can we harness xeno-monitoring techniques to improve post-EPHP surveillance?

The range of ABRs discussed are very broad estimates, covering a wide range of settings, butthis can be a difficult variable to measure consistently. It may be possible to obtain greater certainty in *R_e_* without accurate ABR measures for each location. For example, estimates of low, medium, or high vector densities would still improve our predictions, and these categories of exposure, which act as a proxy for *R*_0_ classification, could be informed by a combination of trap densities and vector-control coverage. Spatial heterogeneity can also occur within **implementation units**, posing problems for any categorization process, so it is important that treatment targets are determined by the maximum transmission measure for a region.

## Concluding Remarks

We have used basic analyses to highlight that the existing experimental evidence does not afford a high degree of certainty at the current 1% mf prevalence elimination threshold. This is mainly because of uncertainties in variables which could be either experimentally or analytically refined, but also due to spatiotemporal variation in vector densities and biting rates [28]. That varying the value of one input variable within sensible ranges found in the literature can make such an impact on predictions, demonstrates the difficulties posed by targeting EOT when we know that local heterogeneities and variability are difficult to measure. Observations of ongoing transmission in parts of validated countries offer empirical support to our concerns with the EPHP target, prompting some important outstanding policy questions (see Outstanding Questions).

In order to support efforts to eliminate LF we would recommend a multipronged approach: improving the experimental evidence base of measurable quantities; detailed analysis of existing infection data to improve our understanding of the infection risk associated with an infectious bite; and development of a discrete system to classify vector density, as a proxy for transmission intensity, to allow comparison of different regions. The optimization of elimination programme strategies and surveillance will require continual revisiting of predictions as we gather more epidemiological data through existing surveys and monitoring infrastructures, as well as expanded epidemiological and surveillance studies at low prevalence.

As more countries cease interventions and move to postvalidation surveillance it is increasingly obvious that transmission breakpoints are unlikely to be one-size-fits-all, hence more flexible thresholds are necessary. It is vital that we ensure that this process is well-informed, as prematurely halting control or surveillance programs could pose a serious threat to global targets, but also because we believe that it may be possible to exploit this geographical variation to maximize the probability of elimination.

## Resources

i www.who.int/lymphatic_filariasis/resources/who_wer9344/en/

ii www.who.int/lymphatic_filariasis/resources/9789241511957/en/

iii www.who.int/lymphatic_filariasis/resources/9789241550161/en/

iv www.who.int/news-room/fact-sheets/detail/lymphatic-filariasis
