# Supplementary material for: Evaluating the Evidence for Lymphatic Filariasis Elimination
Source: Trends Parasitol. 2019 Nov 1;35(12):860–9. doi: 10.1016/j.pt.2019.08.003 (PMC7413036; doi:10.1016/j.pt.2019.08.003)
Supplement: Supplementary file 1 [file TP-2019-j-pt-2019-08-003-S1.docx]

**Supplemental Information**Evaluating the evidence for lymphatic filariasis elimination

Emma L Davis*, University of Warwick

Lisa J Reimer, Liverpool School of Tropical Medicine

Lorenzo Pellis, University of Manchester

T Deirdre Hollingsworth, BDI, Oxford University

*Correspondence: [Emma.Davis@warwick.ac.uk](mailto:Emma.Davis@warwick.ac.uk) (E.L. Davis)

**Box S1. Branching process method details**

*Branching process extinction theory*

The most common branching process formulation is the Galton-Watson process:

Let $X_{n}$ denote the number of infectious individuals in generation$n$ and for each infectious individual, $i$, let $Z_{n,i}$ be the number of new infectious cases directly caused by that individual. $Z_{n,i}$ iid random variables over $n\in\{0,1,2...\}$ and $i\in\{1,...,X_{n}\}$.

Assuming we start a chain of infection with one infectious individual, $X_{0}=1$, we then have the recurrence equation,

$$X_{n+1} = \sum_{i=1}^{X_{n}} Z_{n,i}$$

The extinction probability of one chain of infection is the probability that $X_{n}=0$ for some $n>0$, or that $\lim_{n\to\infty} \mathbb{P[}X_{n}=0]$.

Define $p_{m}$ ($m=0, 1, 2,\ldots$) as the probability of an individual producing $m$ offspring and $d_{m}$ as the probability of extinction by the $m$^th^ generation; $d_{0}=0$ as we start with one individual in generation $0$. Hence $d_{m}$ is an increasing, bounded sequence ($0=d_{0}\leq d_{1}\leq d_{2}\leq... \leq1$) and therefore converges to some limit, $d$, where $0\leq d\leq1$ is the ultimate extinction probability.

$$d_{1}= p_{0}$$

$$d_{2}= p_{0}+\sum_{j=1} p_{j}{{(d}_{1})}^{j}$$

$$\ldots$$

$$d_{m}= p_{0}+\sum_{j=1} p_{j}{{(d}_{m-1})}^{j}$$

We can write this as, $d_{m} = f(d_{m-1})$ where $f$ is the ordinary generating function:

$$f\left( d \right)= p_{0}+\sum_{j=1} p_{j}{(d)}^{j}$$

Since $d_{m}\to d$ as $m\to\infty$, we can find the probability of ultimate extinction by solving $d=f(d)$.

We first need to show that $d$ is the smallest non-negative root of this equation. Take $b>0$ also a root with $b\neq d$ and $b=f(b)$, then we have that $$d_{1}=f(0)\leq f(b)=b$, hence $d_{1}\leq b$. Assume $d_{k}\leq b$ for some *k*, then $d_{k+1}= f(d_{k})\leq f(b) = b$, since $f$ is an increasing function. Hence, by induction $d$ is the smallest non-negative root. The function, $f$, is also convex and hence has at most two real roots. Since one is always a root, $f(1)=\sum_{j=0} p_{j}=1$, then the probability of ultimate extinction is only less than one if the second root both exists and lies between zero and one.

By considering the gradient of $f$ at one, $f^{'}\left( 1 \right)=\sum_{j=1} jp_{j}$, we can determine the location of the other root – namely there is only a second root in $[0,1]$if $f'(1)>1$. Notably this gradient, $f'(1)$, is equal to the average number of secondary cases caused by a single infectious individual, often called the basic reproduction number to describe early outbreak dynamics. Since we are considering a situation where there is a background population prevalence that has been artificially lowered to 1%, we call this the effective reproduction number, $R_{e}$.

Due to the nature of the layered distributions in our model, it isn't possible to directly calculate either the effective reproduction number or the probability of extinction, but both can be calculated numerically by considering the outcome distributions of stochastic simulations. In particular, by calculating the proportion of simulated individual infections that result in each number of onward infections, we can generate a discrete numerical approximation of our secondary case offspring probability distribution.

From this, we can iterate through each generation to find the probability that extinction has occurred. This probability converges over time and, if sufficient generations are considered, can be used as an approximation of the ultimate extinction probability, *d*.

*The simulation process*

Step 1) Choose simulation details:

- Population size
- Number of iterations/simulations to run
- mf prevalence (e.g 1%)
- Choice of parameters (e.g. $k$, $ABR$)

Step 2) Calculate initial set-up:

- Mean worm burden and true prevalence (proportion with $\geq1$ worm); both calculated using aggregation, $k$, and mf prevalence.
- Allocate each individual in the population a bite risk, then distribute the worm burden according to this bite risk

Step 3) For each iteration, calculate for a randomly selected infectious individual:

- Duration of infectious period $\sim$Exponential
- Bite risk $\sim$Gamma (mean $=1$)
- Number of vectors infected over infectious period $\sim$Poisson
- Each vector's lifespan $\sim$Exponential
- Number of vectors that survive incubation $\sim$Binomial
- Number of infectious bites each vector makes $\sim$Poisson

From which we can get the total number of new adult worms, $Y$ established in humans resulting from the entire duration of this one individual's infection (one distinct outcome per iteration, creating a distribution).

Step 4) Calculate total number of new infectious cases:

- Sample $Y$ individuals, with replacement, according to bite risk. Each time an individual is sampled they gain 1 adult worm.
- Compare new worm burdens with previous worm burdens – how many new infectious ($\geq2$ worms) cases are there that were previously not infectious ($\leq1$worm)? This gives our number of secondary infections, $Z$.

Then the mean number of new infectious cases is $R_{e}$. If $R_{e}>1$ then we need to consider the offspring distribution of the branching process, $p_{j}$ probability of having $j$ secondary cases, which can be approximated by the scaled frequency of secondary infectious case counts.

**Evidence for variable ranges**

Table S1 outlines values for biological variables found in the literature, which were used in the calculation of results shown in Figure 4. Values taken as maximum and minimum estimates for our analysis are indicated in bold and mid-value estimates are listed separately. Mid-value estimates that are not also listed in the “Values” column are chosen to represent a mid-ground, usually an average of the maximum and minimum values.

**Table S1. Values for biological variables.**

| Symbol | Description | Values | Mid-value |
| --- | --- | --- | --- |
| $\psi_{1}$ | Prop L3 leave vector per bite | 0.437**(0.363** – **0.511**)[S1], 0.414[S1] | 0.437 |
| $\psi_{2}$ | Prop L3 enter host | 0.223[S2, S3] | 0.223 |
| $w$ | Developmental period in host | **6 months 9 days** *-* **12 months 14 days** [S4], 8 months 4 days[S5], |  |
| $s_{2}$ | Prop L3 develop to adult | (**0.036** *–* **5.68**) x10^-3^[S5-S8], | 0.437 x10^-3^ |
| $b$ | Probability infectious bite infects human | $=\psi_{1}\psi_{2}s_{2}$  (**0.15** *–* **9.29**)x10^-4^  1.47x10^-3^[S5, S8] | 1.43 x10^-4^ |
| $ABR$ | Annual biting rate | **1129**[S9], 1500[S10], 22320[S11], 24090[S9], 26400[S12], 41975[S11], **69120**[S12], 223000[S10] | 24090 |
| $1/g$ | Vector life span (days) | **5.4** *–* **9.5** [S9, S13] | 6.86 |
| $1/r$ | Worm fecund life span (years) | **2.1**[S14], 5.4[S14], 9.1[S15], **11.8**[S15], 40[S16] | 6 |
| $c$ | Probability infectious bite infects vector | 0.37[6] (**0.15** *–* **0.6**)[S13] | 0.37 |
| $v$ | Extrinsic incubation period (days) | *7 – 10* [S7] | 8.5 |
| $f$ | Daily vector blood feeding rate | *0.26 – 0.41* [S13] | 0.335 |
| $k$ | Parasite aggregation | *0.08 – 1* [various] | 0.5 |

**References**

S1. de Meillon, B. et al. (1997) Infection and reinfection of Culex pipiens fatigans with Wuchereria bancrofti and the loss of mature larvae in blood-feeding. Bulletin of the World Health Organization 36 (1), 81.

S2. Norman, R. et al. (2000) EPIFIL: The development of an age-structured model for describing the transmission dynamics and control of lymphatic filariasis. Epidemiology and Infection 124 (3), 529-541.

S3. Ewert, A., Beng C (1967) The fate of Brugia pahangi larvae immediately after feeding by infective vector mosquitoes. Transactions of the Royal Society of Tropical Medicine and Hygiene 61 (5), 659-662.

S4. Addiss, D.G. et al. (2000) Lymphatic Filariasis, World Scientific.

S5. Hairston, N.G. and de Meillon, B. (1968) On the inefficiency of transmission of Wuchereria bancrofti from mosquito to human host. Bulletin of the World Health Organization 38 (6), 935.

S6. Gambhir, M. and Michael, E. (2008) Complex Ecological Dynamics and Eradicability of the Vector Borne Macroparasitic Disease, Lymphatic Filariasis. Plos One 3 (8).

S7. Erickson, S. et al. (2009) Mosquito Infection Responses to Developing Filarial Worms. Plos Neglected Tropical Diseases 3 (10).

S8. Jones, R. (2014) Non-endemic cases of lymphatic filariasis. Tropical Medicine & International Health 19 (11), 1377-1383.

S9. Killeen, G. et al. (2000) A simplified model for predicting malaria entomologic inoculation rates based on entomologic and parasitologic parameters relevant to control. American Journal of Tropical Medicine and Hygiene 62 (5), 535-544.

S10. Michael, E. and Singh, B. (2016) Heterogeneous dynamics, robustness/fragility trade-offs, and the eradication of the macroparasitic disease, lymphatic filariasis. Bmc Medicine 14.

S11. Braack, L. et al. (2015) Biting behaviour of African malaria vectors: 1. where do the main vector species bite on the human body? Parasites & Vectors 8.

S12. Stolk, W. (2005) Lymphatic Filariasis: Transmission, Treatment and Elimination. Journal of Hospital Infection.

S13. Subramanian, S. et al. (1998) The relationship between microfilarial load in the human host and uptake and development of Wuchereria bancrofti microfilariae by Culex quinquefasciatus: a study under natural conditions. Parasitology 116, 243-255.

S14. Vanamail, P. et al. (1996) Estimation of the fecund life span of Wuchereria bancrofti in an endemic area. Transactions of the Royal Society of Tropical Medicine and Hygiene 90 (2), 119-121.

S15. Subramanian, S. et al. (2004) The dynamics of Wuchereria bancrofti infection: a model-based analysis of longitudinal data from Pondicherry, India. Parasitology 128, 467-482.

S16. Carme, B. and Laigret, J. (1979) Longevity of wuchereria-bancrofti var pacifica and mosquito infection acquired from a patient with low-level parasitemia. American Journal of Tropical Medicine and Hygiene 28 (1), 53-55.
